# Randomized trial of Legflow® paclitaxel eluting balloon and stenting versus standard percutaneous transluminal angioplasty and stenting for the treatment of intermediate and long lesions of the superficial femoral artery (RAPID trial): study protocol for a randomized controlled trial

**DOI:** 10.1186/1745-6215-14-87

**Published:** 2013-03-28

**Authors:** Amine Karimi, Sanne W de Boer, Daniël AF van den Heuvel, Bram Fioole, Dammis Vroegindeweij, Jan MM Heyligers, Paul NM Lohle, Otto Elgersma, Rudolf PT Nolthenius, Jan Albert Vos, Jean-Paul PM de Vries

**Affiliations:** 1 Department of Vascular Surgery, St. Antonius Hospital, PO box 2500, Nieuwegein, EM 3430, The Netherlands; 2 Department of Interventional Radiology, St. Antonius Hospital, Koekoekslaan 1, Nieuwegein, CM 3435, The Netherlands; 3 Department of Vascular Surgery, Maasstad Hospital, Maasstadweg 21, Rotterdam, DZ 3079, The Netherlands; 4 Department of Interventional Radiology, Maasstad Hospital, Maasstadweg 21, Rotterdam, DZ 3079, The Netherlands; 5 Department of Vascular Surgery, St. Elisabeth Hospital, Hilvarenbeekseweg 60, Tilburg, GC 5022, The Netherlands; 6 Department of Interventional Radiology, St. Elisabeth Hospital, Hilvarenbeekseweg 60, Tilburg, GC 5022, The Netherlands; 7 Department of Interventional Radiology, Albert Schweitzer Hospital, Albert Schweizerplaats 25, Dordrecht, AT 3318, The Netherlands; 8 Department of Vascular Surgery, Albert Schweitzer Hospital, Albert Schweizerplaats 25, Dordrecht, AT 3318, The Netherlands

## Abstract

**Background:**

Restenosis after percutaneous transluminal angioplasty (PTA) of the superficial femoral artery (SFA) may occur in 45% of patients at 2 years follow-up. Paclitaxel-coated balloons have been found to reduce neointimal hyperplasia, and thus reduce restenosis. Recently, the Legflow® paclitaxel-coated balloon (Cardionovum Sp.z.o.o., Warsaw, Poland) (LPEB) has been introduced. This balloon is covered with shellac, a Food and Drug Administration (FDA) approved natural resin, to obtain an equally distributed tissue concentration of paclitaxel. The RAPID trial is designed to assess restenosis after PTA using the Legflow balloon combined with nitinol stenting versus uncoated balloons with nitinol stenting in SFA lesions >5 cm.

**Methods/Design:**

A total of 176 adult patients with Rutherford class 2 to class 6 symptoms due to intermediate (5–15 cm) or long (>15 cm) atherosclerotic lesions in the SFA will be randomly allocated for treatment with LPEB with nitinol stenting or uncoated balloon angioplasty with stenting. Stenting will be performed using the Supera® stent in both groups (IDEV Technologies Inc., Webster, TX). The primary endpoint is the absence of binary restenosis of the treated SFA segment. Secondary outcomes are target lesion revascularization (TLR), clinical and hemodynamic outcome, amputation rate, mortality rate, adverse events, and device-specific adverse events. Follow up consists of four visits in which ankle-brachial indices (ABI), toe pressure measurements, and duplex ultrasound (DUS) will be performed. Furthermore, a peripheral artery questionnaire (PAQ) will be completed by the patients at each follow-up. In the event that DUS reveals a symptomatic >50% restenosis, or a >75% asymptomatic restenosis, additional digital subtraction angiography will be performed with any necessary re-intervention.

**Discussion:**

The RAPID trial is a multicenter randomized controlled patient blind trial that will provide evidence concerning whether the use of the Legflow paclitaxel/shellac coated balloons with nitinol stenting significantly reduces the frequency of restenosis in intermediate and long SFA lesions compared to standard PTA and stenting.

**Trial registration:**

ISRCTN47846578

## Background

Atherosclerotic lesions in the superficial femoral artery may cause intermittent claudication (IC) and critical limb ischemia (CLI), leading to serious complications such as tissue loss, amputation and even death. Revascularization relieves symptoms and may prevent or delay these complications. Over the last decades, endovascular repair has become the preferred treatment for femoral arterial obstructive disease [1,2]. No definitive consensus has emerged concerning the best endovascular strategy, like the added value of stenting. Literature is most supportive of balloon angioplasty with stenting in longer segment lesions in the superficial femoral artery (SFA). However, even with stenting reported restenosis rates are between 35% and 45% after one and two years follow-up respectively [3,4]. Paclitaxel-coated balloons have been found to reduce restenosis in the Thunder and FEMPAC trials [5,6]. Recently, the Legflow® paclitaxel eluting balloon (Cardionovum Sp.z.o.o., Warsaw, Poland)(LPEB) has been introduced. This paclitaxel eluting balloon is covered with shellac, to obtain an equally distributed tissue concentration of paclitaxel, reaching an optimal dose at a short inflation time of 45 seconds.

So far, no randomized controlled trials have been performed using the Legflow® coated balloon in intermediate and long lesions in the superficial femoral artery combined with primary stenting in both treatment arms.

### Hypothesis

We hypothesize that the Legflow® paclitaxel eluting balloon in combination with nitinol stents will lead to a significantly lower restenosis rate when compared to conventional uncoated balloon angioplasty combined with the same nitinol stents in treatment of intermediate (≥5 cm and <15 cm) and long-segment (≥15 cm) SFA lesions.

## Methods/Design

### Study design

**Figure 1:**
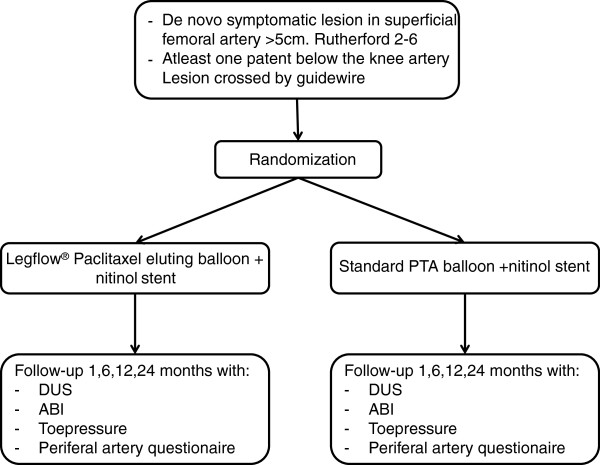
**Flow diagram for inclusion and treatment.** Legend: DUS, duplex ultrasound; ABI, Ankle-Brachial index.

This study is a randomized, controlled, patient-blind, multicenter trial (Figure 1).

### Primary endpoint

The primary endpoint is the absence of binary restenosis at two years. This is defined as the percentage of limbs with absence of hemodynamically significant obstruction in the target lesion after endovascular treatment as indicated below:

• >50% measured as proximal peak velocity ratio (PVR_prox_) ≥2.4 at duplex scanning

• >50% stenosis on digital subtraction angiography (DSA).

### Secondary endpoints

1. Immediate outcome

a. Device success. Defined as exact deployment of the device, according to the instructions for use, using the assigned device only.

b. Technical success. Defined as successful vascular access and completion of the endovascular procedure and immediate morphological success with less than 30% residual diameter reduction and <10 mmHg translesional pressure gradient of the treated lesion on completion.

c. Procedural success. Defined as the combination of technical success, device success and absence of procedural complications.

2. Clinical outcome

a. Distribution of Rutherford stages during follow-up as compared to baseline.

b. In patients with IC: Improvement in claudication onset time (COT) and absolute claudicating time (ACT).

c. Improvement in disease-related health status, functioning and quality of life. As defined by the Peripheral Artery Questionnaire (PAQ).

d. Primary sustained clinical success rate. Defined as improvement by at least one Rutherford category, except for those with actual tissue loss (category 5 and 6), who must at least improve to the level of IC (category 3), in surviving patients, with preserved limb, without the need for target lesion revascularization (TLR).

e. Secondary sustained clinical success rate. Defined as improvement by at least one Rutherford category, expect for those with actual tissue loss (category 5 and 6), who must at least improve to the level of IC (category 3), in surviving patients, with preserved limb, including the need for TLR.

f. Primary sustained resolution of symptoms from peripheral arterial occlusive disease (PAOD) rate. Defined as sustained absence of IC or CLI (Rutherford 0), in surviving patients, with preserved limb, without the need for TLR.

g. Secondary sustained resolution of symptoms from PAOD rate. Defined as sustained absence of IC or CLI (Rutherford 0), in surviving patients, with preserved limb, including the need for TLR.

h. Clinical deterioration rate. Defined as downgrade of more than one category on the Rutherford classification after endovascular treatment (improvements after subsequent TLR/ target-extremity revascularization (TER) are not included).

3. Hemodynamic outcome

a. Mean and median Ankle to Brachial Index (ABI) during follow-up as compared to baseline.

b. Immediate hemodynamic improvement. Defined as post-procedural increase in ABI of ≥0.10 or to an ABI ≥0.9.

c. Primary sustained hemodynamic improvement. Defined as sustained increase in ABI of ≥0.10 or to an ABI ≥0.9, in surviving patients, with preserved limb, without the need for TLR.

d. Secondary sustained hemodynamic improvement. Defined as sustained increase in ABI of ≥0.10 or to an ABI ≥0.9, in surviving patients, with preserved limb, including the need for TLR.

4. Re-occlusion rate. Defined as complete occlusion of the initially treated target-lesion.

5. Target-lesion revascularization (TLR) rate. Defined as the rate and frequency of the need for repeated procedures (endovascular or open surgical) due to a problem arising from the target-lesion (+1 cm proximally and distally to include edge phenomena) in surviving patients with preserved limb. This will be reported as a percentage for each reported frequency (for example, 12% with 1 TLR, 4% with 2 TLR, *etcetera*)

6. Target-extremity revascularization (TER) rate. Defined as the rate and frequency of the need for repeated procedures (endovascular or open surgical) due to a problem arising in the treated limb, but not at the target-lesion site in surviving patients with preserved limb.

7. Mortality rate. Mortality rate associated with the endovascular procedure (that is, mortality within 30 days post-procedure or mortality during a hospitalization >30 days due to the procedure) will be reported separately, as well as overall mortality.

8. Amputation rate. Divided in minor (below the ankle) and major (through or above the ankle). Major amputation is subdivided in below-the-knee, through-knee, and transfemoral. Planned and unplanned amputations will be reported separately. Planned amputations are defined as amputations that were be planned prior to the revascularization procedure (that is, when the revascularization procedure is performed to improve the vascularization (and thereby healing potential) of the planned amputation wound).

9. Rate of device-specific problems (for example, stent fracture, stent migration, balloon rupture).

### Definitions

Definitions of endpoints and parameters are in concordance with the proposed definitions by the Define group, a multidisciplinary team from various specialties involved in PAOD therapies, from Europe and the USA, which has made definitions for more standardized reporting in studies for endovascular treatment of PAOD [7]. There will be a two-year follow-up for all primary and secondary endpoints.

### Patients

A total of 176 patients aged over 18 years, with symptomatic atherosclerotic intermediate or long-segment obstructions of the superficial femoral artery, Rutherford category 2 to 6, will be randomized.

### Eligibility criteria

#### Inclusion criteria

1. Age over 18

2. Symptomatic, atherosclerotic intermediate (≥5 cm and <15 cm) and long (≥15 cm) lesions of the superficial femoral artery.

3. Rutherford class 2 to 6

4. At least one patent below-the-knee artery with uninterrupted flow to the pedal arch.

5. Signed informed consent

6. Randomization will be performed after advancement of a guide wire across the target SFA lesion.

#### Exclusion criteria

1. Life expectancy less than one year.

2. Previous endovascular or surgical treatment of the target superficial femoral artery

3. Inability to comply with the follow-up schedule.

4. Mental disability that hinders the ability to understand and comply with the informed consent.

5. Pregnancy or breast-feeding.

6. Severe renal failure (e-GFR <30 mL/min/1.73 m^2^).

7. Known allergy to iodinated contrast agents.

8. Contra-indication for anti-coagulation (aspirin as well as clopidogrel).

9. (Acute) limb ischemia caused by SFA or popliteal artery aneurysmal disease

10. Obstruction caused by SFA or popliteal artery dissections

### Randomization

Central randomization will be performed by block randomization with use of an automated web-based randomization tool.

### Ethics

This study is conducted in accordance with the principles of the Declaration of Helsinki and Good Clinical Practice guidelines. The study protocol was approved by the Ethics Committee (METC) of the St. Antonius Hospital Nieuwegein (R-12.009). Written informed consent will be obtained from all patients, before randomization.

### Safety and quality control

#### Data safety monitoring board

The Data Safety Monitoring Board (DSMB) is composed of three members: one independent vascular surgeon and two independent interventional radiologists. None of the members is working in one of the hospitals that will include patients. The role of the DSMB is to review safety and to make recommendations regarding the conduct of the study to the steering committee and to the accredited METC that approved the study protocol.

#### Adverse and serious adverse events

Adverse events (AE) are defined as any undesirable experience occurring to a subject during the clinical trial, whether or not considered related to the investigational treatment. All adverse events and serious adverse events reported spontaneously by the subject or observed by the investigator or his staff will be recorded using an adverse events form during admission and throughout 24 months of follow-up. AEs can be classified as moderate or serious and will be recorded on the case record forms (CRFs). A description of the event, start date, end date, whether suspected device-related, any action taken, and the outcome will be described.

A serious adverse event (SAE) is any AE that results in death, or that:

1. results in a life-threatening illness or injury.

2. results in permanent impairment of a body structure or bodily function.

3. requires inpatient hospitalization or prolonged hospitalization.

4. results in medical or surgical intervention to prevent permanent impairment to body structure or bodily function.

Serious adverse events will be classified according to the following four categories:

1. Access complications (may be at the access site or distal to it): Hematoma/bleeding, arterial/venous occlusion/thrombosis, severe vasospasm, intimal injury/dissection, pseudoaneurysm, arteriovenous fistula, vascular perforation or rupture, arterial embolization distal to puncture site.

2. Treatment site complications (may be at the treatment site or proximal or distal to it): Hematoma/bleeding, arterial/venous occlusion/thrombosis, severe vasospasm, intimal injury/dissection, pseudoaneurysm, AV fistula, vascular perforation or rupture, arterial embolization distal to treatment site.

3. Organ-specific complications:

a. Neurological: TIA, minor and major stroke, seizure.

b. Cardiovascular: Hypotension or hypertension requiring treatment, arrhythmia requiring treatment, myocardial ischemia/infarction, chronic heart failure.

c. Respiratory: Profound hypoxia, pulmonary edema, respiratory arrest, pulmonary embolism, pneumothorax.

d. Gastrointestinal: Gastric bleeding, pancreatitis, peritonitis, abscess, perforation of hollow viscus.

4. Systemic complications: Allergic/anaphylactic reaction, renal failure, idiosyncratic reaction to drug, fluid/electrolyte imbalance.

Data on AEs and SAEs will be reported to the DSMB and to the accredited METC via the “Toetsingonline” page of the website of the Central Committee on Research involving Human Subjects (http://www.ccmo.nl).

### Statistical analysis

#### Sample size calculation

Based on the present literature, a two-year restenosis rate of 45% in the standard group and a two-year restenosis rate of 20% in the LPEB group may be expected [3,4]. Given a power of 90% based on a two-tail test with an alpha error level of 0.05, a beta error level of 0.1, an anticipated loss to follow-up of 10%, and a sample size of 88 patients per group, a total of 176 patients will be needed. The analysis will be performed in accordance with intention-to-treat principle. For the primary endpoint, a chi squared test for univariate analysis and Cox proportional hazards model will be assessed to investigate the relation between co-variants and the endpoint. An interim analysis on the primary end point (that is, efficacy) will be at 6 months follow-up of the first 50 consecutive patients by the DSMB. The Peto approach will be followed, meaning that the study will only be stopped for beneficial effects in case of a *P* <0 .001 [8]. The study will not be stopped in case of futility. There will be a blinded outcome assessment by the DSMB for all patients. Missing data will be analyzed using multiple imputation.

### Intervention

The aim of the treatment is to obtain a patent superficial femoral artery, with uninterrupted flow to the pedal arch. Therefore not only the SFA will be treated, but if indicated, the aorto-iliac inflow arteries may be treated additionally during the same procedure. However, for randomization of the SFA stenosis or occlusion, it is mandatory that the inflow artery is treated successfully (that is, a residual obstruction <30% or a translesional mean pressure gradient <10 mmHg). This strategy corresponds with daily practice. Invasive translesional pressure gradients will be measured to determine lesion characteristics.

Before the start of the interventional procedure, a radiopaque ruler must be placed under the leg to measure the length of the lesion. If the common femoral artery (CFA) needs additional treatment, the physician is free to choose a contralateral (cross-over) or ipsilateral (antegrade) technique. At the start of the procedure 40 ml of arterial blood will be drawn from the introducer sheath, just after sheath placement and samples will be collected by the Laboratory of Experimental Cardiology in the context of the Athero Express Biobank Study which has been approved in the past (study nr. C-01.18). DSA images will be made of the ipsilateral limb in at least two planes with a minimum of 30 degrees difference in angulation before and after angioplasty. Radiographic single shots must be made during inflation of the balloons, of the implanted stents, and during post-dilatation of the stents, if performed. All single shots must be stored.

Patients will be randomized to either the LPEB or conventional balloon PTA when the guide wire has successfully passed the SFA lesion. Predilatation with an undersized uncoated PTA balloon will be performed. This will be followed by PTA with LPEB or uncoated balloon, as randomized. To avoid geographic miss this balloon dilatation has to be performed with a longer balloon compared to the length of the lesion itself. Inflation time in either group is at least 45 seconds, allowing adequate drug transfer in case of the LPEB. Next an adequately sized stent (maximum 10% oversizing compared to the diameter of the native SFA below the lesion) is implanted according to instructions for use (IFU). Stenting will be performed using the Supera® wire interwoven nitinol stent (IDEV Technologies Inc., Webster TX). In cases of stenosis including the origin of the SFA, a Smart® stent (Cordis J&J, Bridgewater NJ) will be implanted. The stent must be longer than the treated lesion, but shorter than the balloon that has been used for angioplasty. It is strongly preferred to use one stent to treat the entire SFA lesion. If necessary the stent may be tailored with an additional in-stent balloon dilatation at the discretion of the interventionalist, using a standard (non-drug eluting) PTA balloon of adequate size. A duplex scan of the target lesion must be performed before discharge.

All pre-procedural, procedural, and follow-up imaging studies (magnetic resonance angiogram (MRA), DSA) will be assessed using CAAS QVA 3D (Quantitative Vascular Analysis) software in a core lab.

### Follow-up

All patients receive aspirin 100 mg daily and simvastatin 40 mg daily, indefinitely, starting at least one week prior to the procedure. During the intervention all patients receive 5000 international units of heparin. After the intervention all patients receive additional clopidogrel 75 mg daily for a period of 3 months. Thereafter, aspirin will be continued.

At 1, 6, 12, and 24 months, patients will undergo treadmill test, ABI, toe pressure, and TcpO_2_ measurements of at least the ipsilateral leg. Any complication that occurred within the first 30 days will be recorded. Patients will be asked to fill in a validated peripheral artery questionnaire (PAQ) [9,10]. Furthermore, DUS of the treated SFA will be performed. In case DUS reveals a symptomatic >50% restenosis, or a >75% asymptomatic restenosis additional digital subtraction angiography will be performed with any necessary re-intervention.

### Data collection

Data will be collected by means of an e-CRF during treatment in the participating centers. The CRF will be completed prospectively during hospital admission and follow-up. After that, the CRFs will be forwarded to the data coordinating center. There will be regular contact between the study coordinators and the participating centers.

## Discussion

Prevention of restenosis remains a major challenge in the treatment of peripheral arterial occlusive disease. The use of paclitaxel has proven to inhibit neointimal growth and thus reduce restenosis after percutaneous coronary interventions and SFA lesions [4-6,11]. Recent literature provides evidence that stenting after PTA is of importance to reduce restenosis in intermediate and long-segment SFA lesions [2,3]. The paclitaxel-coated balloons used in previous studies utilize iopromide as excipient, whereas in the current study shellac is the excipient. It is this carrier that distinguishes this balloon from other drug eluting balloons. Shellac might be superior to other excipients in binding the paclitaxel to the balloon and will facilitate a fast and effective delivery of paclitaxel in the arterial wall. In this way lower doses of paclitaxel are needed and shorter inflation time of the balloon itself is required [12]. Furthermore the results of the Thunder and Fempac trials are difficult to interpret on account of the significant heterogeneity in the study populations. Lesions of all lengths were randomized for treatment with paclitaxel-covered balloon or uncoated balloon with bailout stenting using different stents. Furthermore previously treated obstructions and in stent restenosis were included, as well as *de novo* SFA lesions [4,5]. The RAPID study is the first study designed to assess absence of binary restenosis after treatment of SFA lesions >5 cm with a paclitaxel/shellac-coated balloon versus uncoated balloon with stenting in both treatment arms. One of the strengths of this study is that all patients will be treated using the Supera® interwoven nitinol stent (IDEV Technologies Inc., Webster TX), except for lesions involving the origin of the SFA, which will be treated with a Smart stent (Cordis J&J, Bridgewater, NJ). Furthermore the LegFlow® is a recently introduced device that requires a short inflation time of 45 seconds, and may reduce aneurysm formation in the treated segment due to the limited exposure to paclitaxel. Aneurysm formation is described with the use of paclitaxel in drug eluting stents in some case reports. [13-15] Shellac is used in this device to obtain an equally distributed tissue concentration of paclitaxel, reaching an optimal dose at a short inflation time. This results in reduced neointimal growth, and reduces the risk of restenosis. In porcine models paclitaxel/shellac-coated balloons show a higher tissue concentration over time than other paclitaxel balloons, requiring a shorter inflation time for optimal tissue concentrations [12]. Recently, the use of the DIOR 2 balloon (also covered with paclitaxel/shellac and used in the coronary arteries) for in-stent restenosis of small coronary arteries proved safe at one year follow-up. TLR of the treated segments in this study was 12% [7].

The target lesions characterization will be using an anatomical description as advised by the Define group [16]. One of the drawbacks of the current Inter-Society Consensus for the Management of Peripheral Arterial Disease (TASC II) classification is the lack of inclusion of baseline anatomic characteristics of the target (in this trial the SFA) lesion itself and that it combines femoropopliteal lesions as well as below the knee lesions within the same nomenclature [1]. Therefore, the TASC classification, although commonly utilized, may not be ideal. All devices, guide wires and catheters have CE-approval (1434-MDD-32/2011). The shellac used on the catheter is recognized as safe by the FDA (E904).

The RAPID trial is a randomized controlled patient blind trial that will provide evidence of whether the use of paclitaxel/shellac coated balloons with stenting reduces the frequency of restenosis in intermediate (5–15 cm) and long (>15 cm) lesions of the superficial femoral artery.

## Trial status

Approval of the study protocol from the central medical ethical committee has been obtained. Currently 20 patients are enrolled in the RAPID trial. The projected completion date for this trial is August 2015.

## Abbreviations

ABI: Ankle-Brachial Indices; ACT: Absolute claudicating time; AE: Adverse event; CFA: Common femoral artery; CLI: Critical limb ischemia; COT: Claudication onset time; CRF: Case record form; DEB: Drug ellutin balloon; DSA: Digital subtraction angiography; DSMB: Data safety monitoring board; DUS: Duplex ultrasound; FDA: Food and Drug Administration; IC: Intermittent claudication; IFU: Instructions for use; LPEB: Legflow® paclitaxel eluting balloon; MRA: Magnetic resonance angiogram; METC: The ethics committee; PAOD: Peripheral arterial occlusive disease; PAQ: Peripheral Artery Questionnaire; PTA: Percutaneous transluminal angioplasty; PVR: Proximal peak velocity ratio; SAE: Serious adverse event; SFA: Superficial femoral artery; TASC II: Trans-Atlantic Inter-Society Consensus Document on Management of Peripheral Arterial Disease; TER: Target-extremity revascularization; TLR: Target lesion revascularization.

## Competing interests

The authors declare that they have no competing interests.

## Authors’ contributions

AK and JPPMdV drafted the manuscript. AK, JPPMdV, JAV and DAH participated in the design of the study. All authors edited the manuscript and read and approved the final manuscript.
